# Complete Genome Sequences of Actinobacteriophages Anaysia and Caviar

**DOI:** 10.1128/mra.00944-22

**Published:** 2022-10-26

**Authors:** Zain U. Abidin, Joselyne E. Aucapina, Shante Beauzil, Christina M. Berotte, Andrews O. Bonsu, Gabriella Y. Burgos, Solomon Tin Chi Chak, Anaya Collymore, Ethan R. Daley, Raphael Defarias, Veronica Ghobrial, Shivraj S. Gill, Jose M. Huertas-Arias, Hadassah Joseph, Navjot Kaur, Ummay Khan, Connor J. Klein, Horlando Lazo, Yeehen Li, Olivia B. Miller, Javier J. Muñoz, Fernando E. Nieto-Fernandez, Lisa-Marie Nisbett, Darren Owens, Samay M. Patel, Edraine J. Paulino, Samori Pender, Shania M. Perkins, Anjoli Persaud, Tamahina Pierrot, Ibrahim Raja, Kayla L. Riley, Sasha Romero, Paola G. Sarmiento, Kanaja Shorter, Steven Smith, Warda Tahir, Chisom A. Ukekwe

**Affiliations:** a Department of Biological Sciences, SUNY Old Westbury, Old Westbury, New York, USA; DOE Joint Genome Institute

## Abstract

Anaysia and Caviar are temperate siphoviruses isolated from soil using Gordonia terrae 3612 and Mycobacterium smegmatis mc^2^155, respectively. Anaysia’s 52,861-bp genome carries 102 genes, while Caviar’s 47,074-bp genome carries 79 genes. Based on gene content similarity, Anaysia and Caviar are assigned to phage clusters A15 and A3, respectively.

## ANNOUNCEMENT

**FIG 1:**
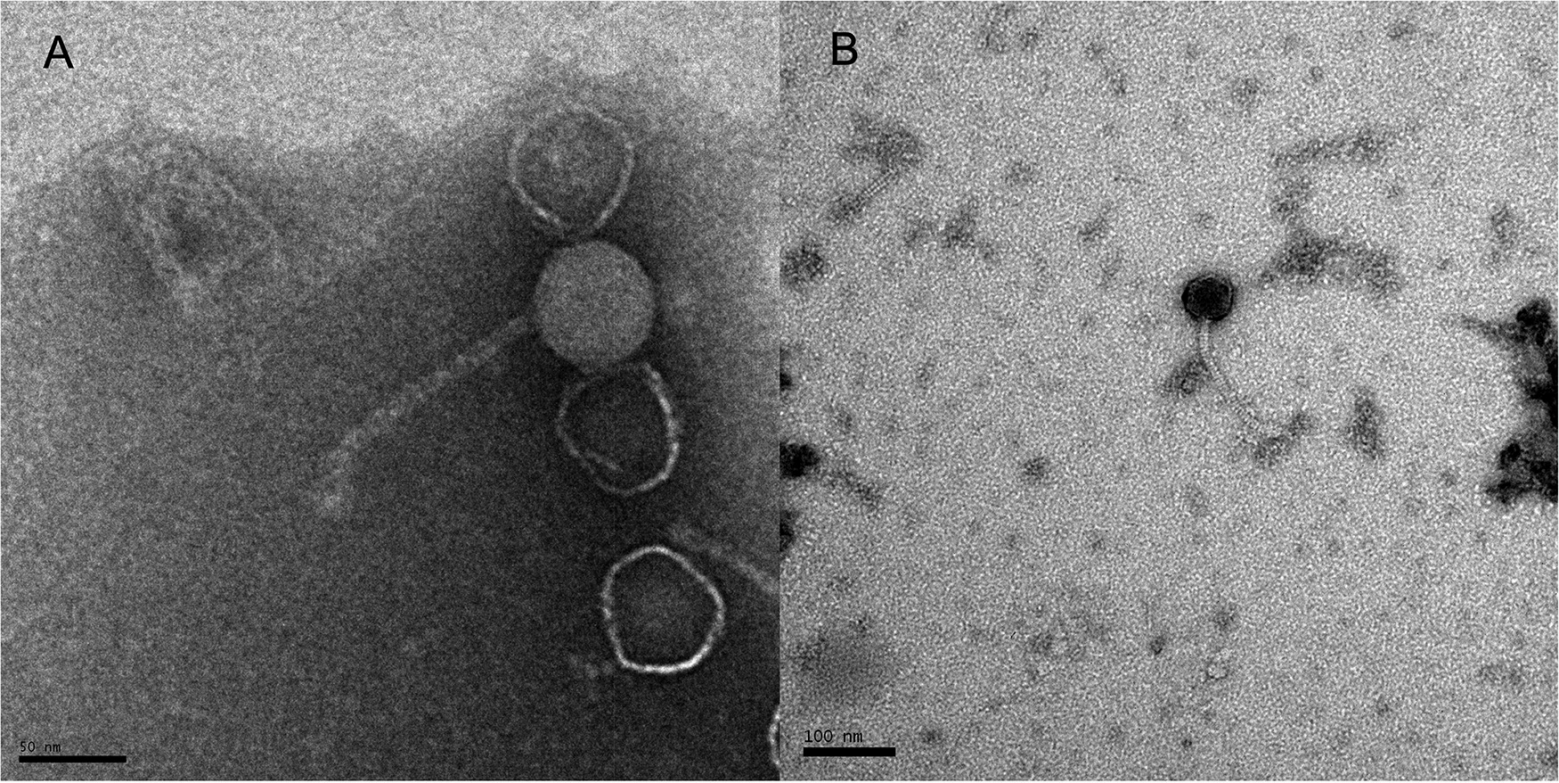
Transmission electron micrographs of Caviar and Anaysia. Phage lysates were mounted onto Formvar-coated copper grids, stained with 1% uranyl acetate, and imaged with a JEOL 1230 instrument. (A) Anaysia, with an icosahedral capsid with a diameter of 55 nm and a tail of 132 nm (*n* = 1). (B) Caviar, with an icosahedral capsid with a diameter of 47 nm and a tail of 164 nm (*n* = 1).

Actinobacteria are a diverse phylum of Gram-positive bacteria of interest due to their medical, agricultural, or biotechnological applications (1–3). The characterization of actinobacteriophages can support the development of molecular tools to genetically manipulate actinobacteria (4, 5). Here, we report the isolation and characterization of two new actinobacteriophages, Caviar and Anaysia, using standard methods (6). Caviar was isolated from grassy marsh soils in Windmill Island in Holland, MI, in 2008 (GPS coordinates 42.800640, −86.096084), whereas Anaysia was isolated from grassy landscaped soil in Mount Saint Mary College, Newburgh, NY, in 2019 (GPS coordinates 41.50467, −74.012878). Briefly, the soil samples that yielded Caviar and Anaysia were washed in 7H9 and peptone-yeast extract-calcium (PYCa) liquid media, respectively, before the wash fluids were collected by centrifugation and filtration (0.02-μm pore size). The resulting filtrates were then inoculated with their respective host bacteria and incubated with shaking for 2 to 5 days at either 30°C (for Gordonia terrae) or 37°C (for Mycobacterium smegmatis). A fraction of each culture was then filtered and plated onto soft agar with its host. After 2 days of incubation, Anaysia forms plaques of 5 to 6 mm in diameter with irregular edges, whereas Caviar forms turbid plaques of 1.5 to 3 mm in diameter. Both phages were purified by 2 rounds of plating. Uranyl acetate-stained transmission electron microscopy images revealed Caviar and Anaysia to be siphoviruses (Fig. 1).

DNA of both phages was extracted from a high-titer lysate using a Promega DNA Wizard kit, prepared as sequencing libraries using a New England BioLabs (NEB) Ultra II library kit, and sequenced on an Illumina MiSeq system (v3 reagents), yielding 325,004 and 66,261 single-end 150-base reads. Raw reads were assembled using Newbler v2.9 and checked for completeness and genomic termini with Consed v29.0 (7). Sequencing data and genomic characteristics are provided in Table 1.

**TABLE 1:** Sequencing data and genome features for Anaysia and Caviar

Phage name	Isolation host	Avg coverage (×)	Genome length (bp)	GC content (%)	Genome termini, sequence	No. of protein-coding genes	No. of tRNA genes
Anaysia	Gordonia terrae 3612	148	52,861	61.9	10-bp 3′ single-stranded overhangs, 5′-CGGGTGGTTA-3′	102	3
Caviar	M. smegmatis mc^2^155	987	47,074	64.2	10-bp 3′ single-stranded overhangs, 5′-CGGCCGGTAA-3′	79	2

Genomes were annotated using DNA Master (http://cobamide2.bio.pitt.edu/computer.htm) embedded with GLIMMER v3.02 (8) and GeneMark v2.5 (9), Starterator v1.2 (https://github.com/SEA-PHAGES/starterator), and PECAAN (10). tRNA and transfer-messenger RNA (tmRNA) were predicted using ARAGORN v1.2.38 (11) and tRNAscan-SE v3.0 (12). Putative functions were assigned using BLAST v2.9 (13), HHpred v3.0beta (14, 15), and Phamerator (16). Default settings were used for all programs.

Totals of 102 and 79 putative genes were identified for Anaysia and Caviar, respectively. Based on gene content similarity (GCS) (https://phagesdb.org/genecontent/) of at least 35% to phages in the actinobacteriophage database, Caviar and Anaysia are assigned to phage clusters A3 and A15, respectively (17, 18). The left halves of both genomes contain structural, assembly, lysis, and tRNA genes that are transcribed rightward, whereas the right halves of both genomes predominantly carry genes involved in DNA metabolism that are transcribed leftward. The tail assembly chaperones in both phages are encoded through programmed translational frameshifts (19). Functions associated with lysogeny can be identified for both phages, including a serine integrase (Caviar gp34), ParA- and ParB-like DNA-partitioning proteins (Anaysia gp36 and gp37), and an immunity repressor (Anaysia gp84), suggesting that both phages are temperate. Interestingly, the region of cluster A3 phage genomes that typically encodes an immunity repressor is lacking in Caviar.

### Data availability.

The genome sequences of Anaysia and Caviar have been deposited in GenBank under accession numbers OP021679 and ON970623, respectively, and in the Sequence Read Archive under accession numbers SRX14443492 and SRX16347218, respectively.
